# Clinicopathological and Targeted Exome Gene Features of a Patient with Metastatic Acinic Cell Carcinoma of the Parotid Gland Harboring an ARID2 Nonsense Mutation and CDKN2A/B Deletion

**DOI:** 10.1155/2015/893694

**Published:** 2015-11-08

**Authors:** Wayne A. Warner, Deborah J. Wong, Fernando Palma-Diaz, Terry Y. Shibuya, Jamil Momand

**Affiliations:** ^1^Division of Oncology, Siteman Cancer Center, Department of Cell Biology and Physiology, Washington University School of Medicine, St. Louis, MO 63110, USA; ^2^Division of Hematology-Oncology, Department of Medicine, University of California, Los Angeles, CA 90095, USA; ^3^Department of Pathology and Laboratory Medicine, University of California, Los Angeles, CA 90095, USA; ^4^Department of Head & Neck Surgery, Southern California Permanente Medical Group, Anaheim, CA 92806, USA; ^5^Department of Otolaryngology-Head & Neck Surgery, University of California Irvine School of Medicine, Orange, CA 92868, USA; ^6^Department of Chemistry and Biochemistry, California State University, Los Angeles, Los Angeles, CA 90032, USA

## Abstract

We describe the presentation, treatment, clinical outcome, and targeted genome analysis of a metastatic salivary acinic cell carcinoma (AciCC). A 71-year-old male presented with a 3 cm right tail of a parotid lesion, first detected as a nodule by the patient seven months earlier. He had a right total parotidectomy with cranial nerve VII resection, right facial nerve resection and grafting, resection of the right conchal cartilage, and right modified radical neck dissection. The primary tumor revealed AciCC with two distinct areas: a well-differentiated component with glandular architecture and a dedifferentiated component with infiltrative growth pattern associated with prominent stromal response, necrosis, perineural invasion, and cellular pleomorphism. Tumor staging was pT4 N0 MX. Immunohistochemistry staining showed pankeratin (+), CD56 (−), and a Ki67 proliferation index of 15%. Upon microscopic inspection, 49 local lymph nodes resected during parotidectomy were negative for cancer cells. Targeted sequencing of the primary tumor revealed deletions of CDKN2A and CDKN2B, a nonsense mutation in ARID2, and single missense mutations of unknown significance in nine other genes. Despite postoperative localized radiation treatment, follow-up whole body PET/CT scan showed lung, soft tissue, bone, and liver metastases. The patient expired 9 months after resection of the primary tumor.

## 1. Introduction

The incidence of salivary gland cancers is approximately 0.6% of the incidence rate of all cancers in the United States [1, 2] and acinic cell carcinomas (AciCCs) account for approximately 2.4% of salivary gland cancers [3]. Most salivary gland AciCCs (86.3%) arise in the parotid gland and, to a much lesser extent, in the submandibular gland, other major and minor salivary glands, the parapharyngeal space, and the sublingual gland [4, 5]. The median age of AciCC presentation is 52 years with 59% incidence in women and 41% incidence in men [5]. Histologically, most tumors tend to be of low grade; however, on occasion, poorly differentiated and high grade tumors occur. The mainstay of treatment entails surgery followed by postoperative radiation [6]. Chemotherapy is generally not considered an effective treatment. The five-year disease-specific survival rate of AciCC is 91% with worse prognosis in cases where there is high grade histology, an age at presentation of greater than 30 years, and evidence for metastatic disease [5]. Local recurrences and distant metastases occur frequently, with most metastases occurring in the lungs and bone and, to a lesser extent, in the central nervous system, mediastinum, and liver [7]. In the single previous mutational spectrum report of a patient with AciCC whole exome analysis revealed deletions in CDKN2A, MTAP, and PPP1R13B along with somatic nonsynonymous point mutations in 14 other genes [8].

We reviewed the medical records and targeted genomic profile of a male who presented with AciCC of the parotid gland. Histopathology and immunohistochemistry were performed at Kaiser Permanente, CA. The patient underwent right total parotidectomy with cranial nerve VII resection, right facial nerve resection and grafting, resection of the right conchal cartilage, and right modified radical neck dissection. Because AciCC of the parotid gland is typically recognized as a low grade and indolent cancer, we sought to further study the genetic profiling of this specific case due to its aggressive clinical nature. Targeted genomic profiling of the parotid tumor using clinical next generation sequencing (NGS) to a minimum coverage depth of 500x was carried out on a FoundationOne platform in a Clinical Laboratory Improvement Amendments (CLIA) certified lab (Foundation Medicine, Cambridge, MA) (Table S1 in Supplementary Material available online at http://dx.doi.org/10.1155/2015/893694) [9]. FoundationOne uses targeted next generation sequencing to interrogate the coding regions of 315 cancer related genes and the introns of 28 genes that are reportedly involved in structural rearrangements (Tables S2 and S3).

## 2. Case Report

### 2.1. Case History

We describe a 71-year-old male who presented with a 2 cm indurated nontender subcutaneous nodule over the right angle of mandible with no redness, warmth, or drainage. According to the patient, the nodule appeared one month earlier and decreased in mass upon eating. His previous medical history was essential hypertension, primary open angle glaucoma, and chronic kidney disease stage 3. One month after presentation, needle aspiration of mass at right tail of the parotid revealed a salivary gland neoplasm. MRI revealed a 2.4 cm anterior-posterior × 2.4 cm transverse × 2.6 cm cranial caudal septated cystic mass in the right parotid lobe. A second fine needle aspiration performed four months after presentation revealed another 1 cm approximate diameter growing mass within the parotid. Five months after presentation, just prior to scheduled surgery, pathology analyses of computed tomography-guided biopsy results were consistent with high grade AciCC. The patient's presurgical cancer workup included complete blood count, coagulation panel, chemistry profile-20, and chest radiographs that were all negative for metastatic cancer.

**Figure 1:**
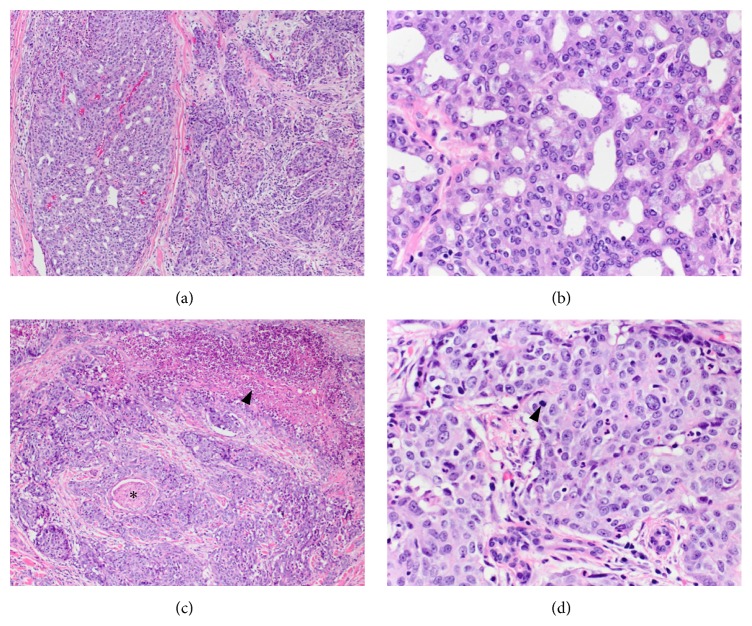
Acinic cell carcinoma of the parotid gland. (a) The tumor exhibits two distinct areas on histologic examination. The two areas are juxtaposed to each other and there is no evidence of transition. On the left, the well-differentiated area shows glandular architecture and the area on the right side of the image shows an infiltrative growth pattern associated with prominent stromal response. H&E, 10x. (b) Higher magnification of the well-differentiated area demonstrates polygonal cells without significant cytologic atypia, variably basophilic granular cytoplasm, minimal pleomorphism, and inconspicuous mitotic activity. H&E, 40x. (c) Dedifferentiated regions of the tumor show focal necrosis (arrowhead) and multiple foci of perineural invasion (star) H&E, 10x. (d) Cytologically, the dedifferentiated areas display more pronounced pleomorphism and increased mitotic activity (arrowhead) H&E, 40x.

The patient underwent a right total parotidectomy with cranial nerve VII resection, right facial nerve resection and grafting, resection of the right conchal cartilage, and right modified radical neck dissection. Intraoperatively, the tumor was noted to encase the right facial nerve and extend to ulcerated overlying skin. The face was rehabilitated with a right AlloDerm facial sling and placement of right upper eyelid gold weight. The facial defect was reconstructed with a cervicofacial advancement flap. Pathology analysis revealed a 3.0 × 3.0 × 3.0 cm AciCC with two distinct areas: a well-differentiated component with glandular architecture and a dedifferentiated component with infiltrative growth pattern associated with prominent stromal response, necrosis, perineural invasion, and cellular pleomorphism (Figure 1). At the time of resection, the staging was pT4, N0, MX. Immunohistochemistry staining showed pankeratin (+), CD56 (−), and a proliferation index of ~15% based on Ki67. The surgical margins were clear with the closest showing tumor 0.2 cm from anterior inked margin.

**Figure 2:**
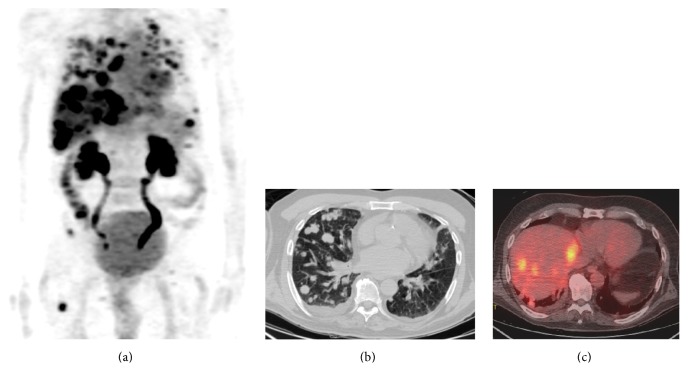
Widespread metastatic acinic cell carcinoma by positron-emission tomography/computed tomography (PET/CT). (a) Whole body PET images demonstrate FDG-avid lesion in the lungs, liver, and bones. (b) Multiple metastatic pulmonary lesions were seen on noncontrast chest CT. (c) Fused PET/CT images demonstrate multiple FDG-avid hepatic metastases.

Postoperatively, the patient received 6000 cGy in 30 fractions using intensity modulated radiation therapy (IMRT) to the parotid bed and right neck. Three months after completing IMRT, a whole body PET/CT scan revealed multiple fluorodeoxyglucose- (FDG-) avid pulmonary nodules, a 2.3 cm mass in the right infrahilar region with a maximum standardized uptake value (SUV) of 5.74, a 2 × 2.6 cm subcarinal lymph node (SUV 6.08), multiple FDG-avid liver lesions, a soft tissue lesion in the lateral left upper quadrant 3 × 2.7 cm, and osseous metastases to the tenth thoracic vertebrae (T10) (maximum SUV 8.4) and proximal right femur (Figure 2). The patient developed dermal metastases along the neck incision site. A CT-guided biopsy of a pulmonary nodule confirmed metastatic AciCC with immunohistochemistry negative for thyroid transcription factor-1 (TTF-1), cytokeratin 7 (CK7), and cytokeratin 20 (CK20).

The patient underwent palliative radiotherapy to a painful left calcaneal bone metastasis and right femur metastasis. He was placed on zoledronic acid for the osseous metastasis. He was seen by medical oncology to discuss systemic treatment options, but the patient declined the treatment. He was ultimately placed on hospice due to progressive metastatic disease and worsening functional status.

### 2.2. Genomic Profiling

**Figure 3:**
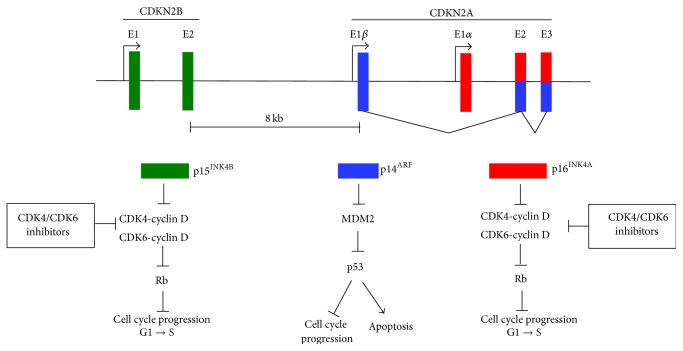
The CDKN2A-CDKN2B locus regulates the cell cycle. Genomic organization of the 9p21 locus containing CDKN2B encoding p15^INK4B^ and CDKN2A encoding p14^ARF^ and p16^INK4A^. p14^ARF^ is coded by exons E1*β*, E2, and E3 and p16^INK4A^ is coded by exons E1*α*, E2, and E3. E2 and E3 are shared by p14^ARF^ and p16^INK4A^, but they use alternate reading frames to produce unique protein sequences. p15^INK4B^ and p16^INK4A^ induce cell cycle arrest by complexing to CDK4 and CDK6 and preventing their complex formation with cyclin D. Rb remains active (dephosphorylated) and prevents E2F-mediated transcription of target genes necessary for cell cycle progression from G1 to S phase. p14^ARF^ inhibits cell cycle progression and activates apoptosis by inhibiting MDM2, which leads to activation of p53.

The NGS analysis reported deletions of two tumor suppressor genes on chromosome 9p21 that encode three tumor suppressor proteins and a nonsense mutation in another tumor suppressor gene on chromosome 12q12 (Table 1). One tumor suppressor gene is CDKN2B (cyclin-dependent kinase inhibitor 2B), which encodes p15^INK4B^ [10–13] (Figure 3). The second tumor suppressor gene is CDKN2A, which encodes two proteins, p16^INK4A^ and p14^ARF^. p16^INK4A^ and p15^INK4B^ are CDK inhibitors that separately form complexes with cyclin-dependent kinase 4 (CDK4) and cyclin-dependent kinase 6 (CDK6) to inhibit their interaction with cyclin D. These CDK inhibitors induce cell cycle arrest at G1 checkpoints, preventing phosphorylation of retinoblastoma protein (Rb), thus halting the cell cycle. p14^ARF^ is created from a unique splicing event and uses alternative reading frame in exons 2 and 3 of CDKN2A. p14^ARF^ stabilizes the tumor suppressor p53 by inhibiting its negative regulator MDM2. Deletion of CDKN2A leads to loss of p14^ARF^, which causes abrogation of p53-dependent cell cycle arrest, apoptosis, and other tumor suppressor functions [14, 15].

**Figure 4:**
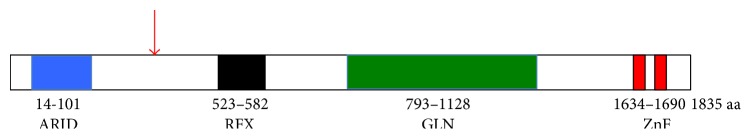
The protein domain arrangement in ARID2 protein. ARID2 has four discrete domains: the ARID domain (residues 14–101), the RFX domain (residues 523–582), the GLN domain (residues 793–1128), and a domain that contains two ZnFs (1634–1690). The red arrow indicates the approximate location of the E267^*∗*^ nonsense mutation identified in the AciCC of the patient.

Another tumor suppressor gene mutated in the patient's AciCC was AT-Rich Interactive Domain 2 (ARID2), which encodes AT-Rich Interactive Domain 2 protein (ARID2 protein), also known as BRG1-associated factor 200 (BAF200). ARID2 protein is a subunit of the polybromo- and BRG1-associated factor chromatin remodeling complex (PBAF) and appears to be necessary for complex stability [16]. In general, PBAF is responsible for nuclear receptor ligand-dependent transcriptional activation and is part of the SWI/SNF family of chromosome remodeling proteins [17]. A lack of ARID2 protein alters cellular transcription response to *α*-interferon [16] and inhibits osteoblast differentiation [18]. ARID2 protein has four domains: AT-rich interaction domain (ARID), RFX-like DNA binding motif (RFX), proline- and glutamine-rich region (GLN), and two C-terminal C2H2 zinc finger motifs (ZN) [19] (Figure 4). The patient harbored a loss-of-function (LoF) nonsense E267^*∗*^ mutation resulting in a truncated protein deficient in the RFX, GLN, and ZN.

Nine missense mutations of unknown significance in AciCC were identified in AXL, ESR1, FBXW7, GNA13, KEAP1, KLHL6, LRP1B, PLCG2, and PRSS8 (Table 1). Other mutations in these nine genes have been reported in many cancer types underscoring the need for functional genomics to ascertain the significance of these particular changes in AciCC [20]. Interestingly, 9 of the 12 documented genomic insults are in genes involved in signaling pathways or regulation of the cell cycle (Table 1). Of the 10 point mutations observed in the sample, 8 were transversions.

## 3. Discussion

The case presented herein is of a highly aggressive AciCC in which both local recurrence and distant metastatic disease occurred within six months of curative intent surgery and adjuvant radiotherapy. Histologically, the presence of dedifferentiation was a poor prognostic feature. Furthermore, we detected two components juxtaposed to one another; one is well-differentiated with glandular architecture and the second shows infiltrative growth pattern associated with stromal response. The second area has dedifferentiated regions with focal necrosis, multiple foci of perineural invasion, pronounced pleomorphism, and increased mitotic activity. Interestingly, the juxtaposition of the two areas is a feature found in a subset of AciCC (*n* = 25) that failed to respond to extensive resection and adjuvant therapy [21]. In this subset, local recurrence or distant metastasis occurred in most patients with 72.7% dead with disease with a mean of 2.9 years.

In addition, targeted next generation sequencing revealed loss of tumor suppressor genes, CDKN2A, CDKN2B, and ARID2, as well as several mutations in genes involved in cell signaling, ubiquitination, cell cycle regulation, and endocytosis. The presence of the multitude of genetic alterations underscores the high grade, poorly differentiated nature of the tumor and, conversely, may help to explain its highly aggressive nature. Of course, because of the targeted nature of this analysis, it is quite possible that there are mutations in other critical genes not analyzed.

A single other report has been published where extensive genetic analysis of a salivary gland AciCC primary tumor was conducted [8]. In that report, whole exome sequencing analysis was performed. A comparison with our targeted sequencing study may shed light on features shared in the two cases. It was reported that a 58-year-old woman presented with a right parotid mass consistent with Warthin's tumor. An initial CT scan revealed a right parotid mass. A year later, another CT scan showed a mass centered in the right parotid effacing the jugular vein and abutting the mandible and skull base with extension along the facial nerve to the geniculate ganglion. Pathologic examination revealed a low grade AciCC with extensive perineural and lymphovascular spread and the lymph nodes negative for malignancy. In some respects, this contrasts with our patient who had a high grade AciCC and developed multiple metastases. However, similar to the previous case, the lymph nodes of our patient were negative. The previous case study reported that 35 genes were identified with somatic copy number aberrations and 14 with somatic single nucleotide variations. Somatic deletion of CDKN2A was the only genomic aberration detected in both patients, although we must consider that in our study only a fraction of the exome was analyzed.

In both salivary gland AciCC tumors, the CDK4/6/Rb and p53 pathways were targeted. It is worth noting that deletion of CDKN2A is a frequent event in heat and neck tumors. In one report, 22% of head and neck tumors (*n* = 279) have a deletion in CDKN2A [22]. Of the 27 active clinical trials underway for CDK4/6 inhibitors, four are specific for solid tumors with CDK4/6 pathway activation [23]. The FDA recently approved the CDK4/6 inhibitor palbociclib for treatment of metastatic breast cancer in combination with the aromatase inhibitor, letrozole [24]. In cancers that have genomic driver insults in CDKN2A or CDKN2B, inhibition of CDK4/6 is likely to be a therapeutic option worth investigating.

The other clinical target relevant to this study is MDM2 [25]. MDM2 binds to the p53 transactivation domain and mediates polyubiquitination of p53, leading to p53 proteolysis by the 26S proteasome [26]. Seven drugs that prevent complex formation between MDM2 and p53 are in phase I clinical trials. Because the drugs will also likely activate p53 in normal cells, it is expected that some side effects will occur. Indeed, early results from clinical trials show that two MDM2 targeting drugs cause thrombocytopenia and one drug causes apoptosis of megakaryocyte progenitor cells [25, 27, 28]. Other approaches to identify MDM2 inhibitors without severe side effects are being explored [29].

This case study is the first report of the ARID2 nonsense mutation (E267^*∗*^) in AciCC. Two other cases with this specific genomic insult (AA E267^*∗*^; nuc c799G>T) have been reported in hepatocellular carcinoma [30, 31]. A recent study revealed that 18.2% of patients with HCV-associated hepatocellular carcinoma have inactivating mutations in ARID2 [32]. ARID2 mutations have also been reported in pancreatic cancer, colorectal cancer, melanoma, and non-small-cell lung cancer [32–42]. Furthermore, in 60 hepatocellular samples evaluated by whole exome or genome sequencing, 35% harbored alterations in chromatin remodeling genes, including ARID1, another member of the ARID superfamily [32]. A study identified ARID2 loss-of-function mutations in 5% of non-small-cell lung cancers, making it the 6th most frequently mutated genes in this cancer type after TP53, KRAS, EGFR, CDKN2A, and STK11 [35].

In our AciCC case, we also observed missense mutations in AXL, ESR1, FBXW7, KEAP1, KLHL6, GNA13, PLCG2, and LRP1B. Mutations in these genes have been reported in non-head and neck cancers [30, 31]. AXL has been implicated in epithelial-mesenchymal transition (EMT) and has been hypothesized to play a key role not only in development of metastasis, but also in resistance to chemotherapy and targeted therapies, including lapatinib resistance in HER2-positive breast cancer [43] and in erlotinib resistance in EGFR-mutant non-small-cell lung cancer [44]. Among other salivary gland neoplasms, genomic alterations in FBXW7, KLHL6, and LRP1B have been identified in adenoid cystic carcinoma [33, 45]. Alterations of the oxidative stress gene KEAP1 was reported in human papilloma virus negative head-and-neck squamous cell carcinomas (HNSCCs) (5%) [46].

FBXW7 codes for F-box and WD40 repeat domain containing 7 protein (Fbxw7), a substrate recognition component of the SCF (Skp1-Cul1-F-box protein) type ubiquitin ligase complex. Fbxw7 plays a critical role in cell division, growth and differentiation by binding to proto-oncoproteins (c-Myc, Notch1, Notch4, c-Jun, cyclin E, KLF5, and mTOR) [47]. The binding domain within Fbxw7 is a conserved phosphorylated domain known as the Cdc4 phosphodegron and leads to ubiquitin-dependent proteolysis of the above proto-oncoproteins. Since most of its substrates are growth-promoting, Fbxw7 is thought of as a tumor suppressor. Overall, approximately 6% of multiple samples from 15 different human tumor types surveyed harbor FBXW7 mutations [48]. Most of these mutations result in amino-acid substitutions at key positions in the WD40 repeats with consequential disruption of substrate binding. Hot spot mutations were observed in the third WD40 repeat (nucleotides 1,393, 1,394 and 1,436). The remaining mutations were either nonsense mutations or mutations of unknown significance. The tumor in our case study harbored one of these mutations of unknown significance, E192A. A fibrolamellar HCC patient harboring the same mutation was treated with vorinostat and sirolimus as part of a phase I clinical trial for mTOR inhibitors. The time to treatment failure was 6.8+ months [49]. Thirty-one percent of T cell acute lymphocytic leukemia (T-ALL) patients have FBXW7 mutations with one reported case harboring the E192A mutation [50].

In this report, targeted next generation sequencing was performed on the excised primary parotid tumor. Recently, researchers used whole genome sequencing data to develop and validate “mutant-allele tumor heterogeneity” (MATH) as a measure of intratumor heterogeneity in HNSCC suggesting that it should be considered as a biomarker for survival in these cancers [51]. Given that high intratumor heterogeneity leads to worse clinical outcomes, one limitation of this study is that repeated sampling of the tumor with whole exome sequencing was not performed.

Recent cancer sequencing studies have unleashed an ever-expanding catalog of somatic aberrations, involving novel genes and pathways in many cases [46, 52–56]. This extensive mutational heterogeneity can inform genome forward treatment algorithms. This case study underscores the need to sequence more AciCC samples multiple times for the genomic, transcriptome, epigenomic, and proteomic annotation of molecular alterations. Such information will deepen our understanding of the pathophysiology, risk stratification, prognosis of AciCC, and guide precision genome forward treatment.

## Supplementary Material

Table S1 of Supplementary Material presents the sequencing pipeline performance specifications and quality metrics. Table S2 lists the 315 genes (exons only) assayed by Foundation One. Table S3 lists the introns of 28 genes commonly involved in rearrangements assayed by Foundation One. These genes were confirmed to be somatically compromised in human solid tumors as evidenced by their validation for therapy (approved or clinical trials), and/or reported as unambiguous drivers of oncogenesis.

## Figures and Tables

**Table 1:** Somatic genomic alterations.

Gene	Chr	Coding change	Base change	Pathway
CDKN2A	9	Loss	Loss	Cyclin-dependent kinase inhibitor, Rb tumor suppressor
CDKN2B	9	Loss	Loss	Cyclin-dependent kinase inhibitor, Rb and p53 tumor suppressors
ARID2	12	E267^*∗*^	799G>T	Chromatin remodeling, tumor suppressor
AXL	19	T112M	335C>T	Receptor tyrosine kinase signaling
ESR1	6	G90R	268G>C	Transcription factor
FBXW7	4	E192A	575A>C	Phosphorylation-dependent ubiquitination
GNA13	17	T365S	1093A>T	G-protein signaling
KEAP1	19	C624Y	1871G>C	Sensor of oxidative stress
KLHL6	3	S497T	1490G>C	Receptor signaling
LRP1B	2	N1724Y	5170A>T	Receptor-mediated endocytosis signaling
PLCG2	16	F528C	1583T>G	Transmembrane signaling enzyme
PRSS8	16	A209T	625G>A	Proteolytic enzymes
