# Supplementary material for: Clinicopathological and Targeted Exome Gene Features of a Patient with Metastatic Acinic Cell Carcinoma of the Parotid Gland Harboring an ARID2 Nonsense Mutation and CDKN2A/B Deletion
Source: Case Rep Oncol Med. 2015 Nov 8;2015:893694. doi: 10.1155/2015/893694 (PMC4655020; doi:10.1155/2015/893694)
Supplement: Supplementary file 1 — Table S1 of Supplementary Material presents the sequencing pipeline performance specifications and quality metrics. Table S2 lists the 315 genes (exons only) assayed by Foundation One. Table S3 lists the introns of 28 genes commonly involved in rearrangements assayed by Foundation One. These genes were confirmed to be somatically compromised in human solid tumors as evidenced by their validation for therapy (approved or clinical trials), and/or reported as unambiguous drivers of oncogenesis. [file 893694.f1.pdf]

Clinicopathological and Targeted Exome Gene Features of a Patient with Metastatic Acinic Cell Carcinoma of the Parotid Gland Harboring a ARID2 Nonsense Mutation and CDKN2A/B Deletion

Supplementary Materials

Table S1| Sequencing specifications.

	Base Substitutions	Indels	Copy Number Alterations	Rearrangements
Sensitivity	>99%<br>MAF ≥5%	>97%<br>MAF ≥10%	≥95%<br>CN≥8 or 0<br>≥ 30% tumor nuclei	≥90%**<br>>99% for ALK fusion***<br>≥ 20% tumor nuclei
Specificity (PPV)	>99%	>99%	>99%	
Typical median depth of coverage*	500****			

*Each covered read is of a unique DNA fragment to enable detection of alterations at low frequency [57]. **Based on analysis of coverage and re-arrangement structure in the COSMIC database for solid tumor fusion genes where alteration prevalence could be established, complemented by detection of exemplar rearrangements in cell line titration experiments [57]. ***Based on ALK re-arrangement concordance analysis vs. a standard clinical FISH assay [57]. ****[9]. MAF, mutant allele frequency; CN, copy number; PPV, Positive Predictive Value.

Table S2|Genes assayed by FoundationOne®.

ABL1	CCND2	ERBB2	GATA6	KMT2C (MLL3)	NTRK2	RNF43	TP53
ABL2	CCND3	ERBB3	GID4 (C17orf39)	KMT2D (MLL2)	NTRK3	ROS1	TSC1
ACVR1B	CCNE1	ERBB4	GLI1	KRAS	NUP93	RPTOR	TSC2
AKT1	CD274	ERG	GNA11	LMO1	PAK3	RUNX1	TSHR
AKT2	CD79A	ERRFI1	GNA13	LRP1B	PALB2	RUNX1T1	U2AF1
AKT3	CD79B	ESR1	GNAQ	LYN	PARK2	SDHA	VEGFA
ALK	CDC73	EZH2	GNAS	LZTR1	PAX5	SDHB	VHL
AMER1 (FAM123B)	CDH1	FAM46C	GPR124	MAGI2	PBRM1	SDHC	WISP3
APC	CDK12	FANCA	GRIN2A	MAP2K1	PDCD1LG2	SDHD	WT1
AR	CDK4	FANCC	GRM3	MAP2K2	PDGFRA	SETD2	XPO1
ARAF	CDK6	FANCD2	GSK3B	MAP2K4	PDGFRB	SF3B1	ZBTB2
ARFRP1	CDK8	FANCE	H3F3A	MAP3K1	PDK1	SLIT2	ZNF217
ARID1A	CDKN1A	FANCF	HGF	MCL1	PIK3C2B	SMAD2	ZNF703
ARID1B	CDKN1B	FANCG	HNF1A	MDM2	PIK3CA	SMAD3	
ARID2	CDKN2A	FANCL	HRAS	MDM4	PIK3CB	SMAD4	
ASXL1	CDKN2B	FAS	HSD3B1	MED12	PIK3CG	SMARCA4	
ATM	CDKN2C	FAT1	HSP90AA1	MEF2B	PIK3R1	SMARCB1	
ATR	CEBPA	FBXW7	IDH1	MEN1	PIK3R2	SMO	
ATRX	CHD2	FGF10	IDH2	MET	PLCG2	SNCAIP	
AURKA	CHD4	FGF14	IGF1R	MITF	PMS2	SOCS1	
AURKB	CHEK1	FGF19	IGF2	MLH1	POLD1	SOX10	
AXIN1	CHEK2	FGF23	IKBKE	MPL	POLE	SOX2	
AXL	CIC	FGF3	IKZF1	MRE11A	PPP2R1A	SOX9	
BAP1	CREBBP	FGF4	IL7R	MSH2	PRDM1	SPEN	
BARD1	CRKL	FGF6	INHBA	MSH6	PREX2	SPOP	
BCL2	CRLF2	FGFR1	INPP4B	MTOR	PRKAR1A	SPTA1	
BCL2L1	CSF1R	FGFR2	IRF2	MUTYH	PRKCI	SRC	
BCL2L2	CTCF	FGFR3	IRF4	MYC	PRKDC	STAG2	
BCOR	CTNNA1	FGFR4	IRS2	MYCL (MYCL1)	PRSS8	STAT3	
BCORL1	CTNNB1	FH	JAK1	MYCN	PTCH1	STAT4	
BLM	CUL3	FLCN	JAK2	MYD88	PTEN	STK11	
BRAF	CYLD	FLT1	JAK3	NF1	PTPN11	SUFU	
BRCA1	DAXX	FLT1	JUN	NF2	QKI	SYK	
BRCA2	DDR2	FLT4	KAT6A (MYST3)	NFE2L2	RAC1	TAF1	
BRD4	DICER1	FOXL2	KDM5A	NFKBIA	RAD50	TBX3	
BRIP1	DNMT3A	FOXP1	KDM5C	NKX2-1	RAD51	TERC	
BTG1	DOT1L	FRS2	KDM6A	NOTCH1	RAF1	TERT*	
BTK	EGFR	FUBP1	KDR	NOTCH2	RANBP2	TET2	
C11orf30 (EMSY)	EP300	GABRA6	KEAP1	NOTCH3	RARA	TGFBR2	
CARD11	EPHA3	GATA1	KEL	NPM1	RB1	TNFAIP3	
CBFB	EPHA5	GATA2	KIT	NRAS	RBM10	TNFRSF14	
CBL	EPHA7	GATA3	KLHL6	NSD1	RET	TOP1	
CCND1	EPHB1	GATA4	KMT2A (MLL)	NTRK1	RICTOR	TOP2A	

Assay interrogated the entire coding sequence of 315 cancer-implicated genes that are

druggable targets (validated or in clinical trials), established oncogenic drivers and/or somatically compromised in solid tumors. *Promoter only

Table S3| Select rearrangements assayed by FoundationOne®.

ALK	BRCA1	ETV1	FGFR2	MYB	NTRK2	RET
BCL2	BRCA2	ETV4	FGFR3	MYC	PDGFRA	ROS1
BCR	BRD4	ETV5	KIT	NOTCH2	RAF1	TMPRSS2
BRAF	EGFR	ETV6	MSH2	NTRK1	RARA	

Assay interrogated selected introns from 28 genes structurally rearranged in solid tumors.
